# “Clinical triad” findings in pediatric Klippel-Feil patients

**DOI:** 10.1186/s13013-016-0075-x

**Published:** 2016-06-27

**Authors:** Dino Samartzis, Prakasam Kalluri, Jean Herman, John P. Lubicky, Francis H. Shen

**Affiliations:** Department of Orthopaedics and Traumatology, Queen Mary Hospital, The University of Hong Kong, Hong Kong, SAR People’s Republic of China; Colonial Orthopedics, Colonial Heights, VA USA; Shriners Hospitals for Children, Chicago, IL USA; Department of Orthopaedic Surgery & Pediatrics, West Virginia University School of Medicine, Morgantown, WV USA; Department of Orthopaedic Surgery, University of Virginia, Charlottesville, VA USA

**Keywords:** Klippel-Feil, Cervical, Spine, Congenital, Fusion, Clinical, Triad, Epidemiology, Classification

## Abstract

**Background:**

It has been propagated that patients with Klippel-Feil syndrome (KFS) exhibit “clinical triad” findings (CTFs), known as a short neck, low posterior hairline, and limited cervical range of motion (ROM). However, the literature has noted that up to 50 % of KFS cases may not present with such findings and the reasoning behind such assertions remains speculative. As such, the following study addressed the association between CTFs to that of congenitally-fused cervical segments and other risk factors in KFS patients.

**Methods:**

We conducted a retrospective clinical study based on prospectively collected radiographic data. Thirty-one KFS patients at a single institution were assessed. Radiographs were used to evaluate the location and extent of congenitally-fused segments (spanning the occiput (O) to the first thoracic vertebra (T1)), as well as examining coronal and sagittal cervical alignments based on the Samartzis et al. KFS classification. Clinical records were evaluated to account for the initial clinical assessment of CTFs. Patients were further stratified into two groups: Group 1 included patients noted to have any CTFs, while Group 2 included patients who had no such findings.

**Results:**

There were 12 males and 19 females (mean age at initial consultation: 9.7 years). No evidence of any of the CTFs was shown in 35.5 % of patients, whereas 38.7, 16.2 and 9.7 % were determined to have one, two or all three criteria, respectively. Limited cervical ROM was the most common finding (64.5 % of patients). In Group 1, 25 % had a short neck, 30 % a low posterior hairline, and 100 % exhibited limited cervical ROM. Group 1 had a mean of 3.9 fused cervical segments, whereas Group 2 had a mean of 2.5 fused cervical segments (*p* = 0.028). Age, sex-type, occipitalization and alignment parameters did not significantly differ to Group-type (*p* > 0.05). In Group 1, based on the Samartzis et al. Types I, II, and III, 16.7, 73.3, and 80.0 % of the patients, respectively, had at least one CTF.

**Conclusions:**

Complete CTFs were not highly associated during the clinical assessment of young KFS patients. However, KFS patients with extensive, congenitally-fused segments (i.e. Samartzis et al. Type III) were significantly more likely to exhibit one of the components of the CTF, which was predominantly a limited cervical ROM. Clinicians managing young pediatric patients should not rely on the full spectrum of CTFs and should maintain a high-index of suspicion for KFS, in particular in individuals that exhibit associated spinal findings, such as congenital scoliosis.

## Background

Klippel-Feil syndrome (KFS) is a complex condition, noted as improper segmentation or congenital fusion of at least one vertebral motion segment of the cervical spine with or without additional spinal or extraspinal manifestations [1–4]. Studies have shown that extensive congenital fusion of the cervical spine is associated with improper formation of the vertebral segments and altered biomechanics, often leading to vertebral maldevelopment, degenerative manifestations, hypermobility and instability, neurologic compromise, and potential for severe neurologic injury [4–18]. As such, early diagnosis of KFS is imperative in identifying high-risk cases and design measures to prevent potentially fatal outcomes.

It has been propagated that KFS patients exhibit the “clinical triad” findings, known as a short neck, low posterior hairline, and a limited cervical range of motion (ROM), which aids in the identification of this syndrome [2, 3]. However, studies have noted that up to 50 % of KFS cases may not present with such findings [1, 19, 20]. Nevertheless, the reasoning behind such observations and the disconnect from the classic description of the clinical triad has remained speculative.

It is believed that KFS occurs in 1 out of 42,000 births; [21–24] however, the true incidence of this condition has not been properly assessed and may vary between populations [1, 25]. Such a condition may be under-reported due to a lack of prompt clinical identification, which may be attributed to the criteria associated with the clinical triad signs that have been propagated throughout the years to guide the identification of KFS patients. Previous studies of KFS patients have noted that the congenital fusion process affects vertebral growth, and that motion at the upper and lower cervical spine region is also diminished due to improper segmentation of the vertebral segments [11, 12, 19, 26]. Therefore, the authors hypothesize that the extent of congenitally-fused cervical segments may affect the phenotype of the clinical triad associated with KFS. As such, the following study examined KFS patients from a single institution to address the co-existence of congenitally-fused cervical segments and clinical triad findings.

## Methods

### Study population

The study was a retrospective chart review based on imaging data collected prospective of patients with KFS who were assessed at the orthopaedic clinic at the Shriners Hospitals for Children in Chicago, Illinois between 1986 and 2004. Following Institutional Review Board study approval, 31 consecutive patients were identified that presented with complete clinical and radiographic records available for evaluation. Patients initially sought consultation and assessment for the following reasons: syndromic work-up of their condition, required pre-anesthesia clearance for non-cervical spine surgery, or referral by a physician not related to the institute.

### Imaging assessment

Radiographically, congenital fusion of the cervical spine from C1 to T1 was noted by trabecular bone bridging and loss of motion as evidenced upon anteroposterior and lateral neutral, flexion, and extension plain radiographs. Occipitalization (O-C1) was regarded as congenital fusion of the occiput to the atlas. The location and extent of congenital fusion as well as alignment parameters were assessed by individuals (DS, PK, FHS) blinded to the clinical assessment of each patient. Patients were regarded as having KFS if at least one motion segment of the cervical spine exhibited congenital fusion. The Samartzis et al. [7] classification scheme of congenitally-fused segments in KFS patients was implemented and entailed the following: (a) Type I: single block, congenitally-fused segment; (b) Type II: multiple, non-contiguous congenitally-fused segments; and (c) Type III: multiple, contiguous congenitally-fused segments. The cervical spine was further stratified into upper (O-C2), mid (C2-C4), and lower (C4-T1) vertebral levels and the presence of fused segments within those regions. The coronal cervical alignment was assessed and defined as the resultant of the intersecting lines from C1 and the inferior border of C7. Similarly, the sagittal cervical alignment was measured by the intersecting lines from C1 and the inferior border of C7. The measuring tools remained consistent for each patient.

### Clinical assessment

Clinical charts were assessed to account for physician interpretation of clinical findings based on the initial patient consultation. Patients were clinically assessed by spine fellowship trained orthopaedic surgeons. Findings of any component of the clinical triad (i.e. short neck, low posterior hairline, limited cervical ROM) were recorded. To account for potential physician assessment bias, our sample size was further stratified into two Groups: Group 1: patients noted to have any “clinical triad” findings; and Group 2: patients with no “clinical triad” findings. Additional patient demographics (i.e. age, gender) and risk factors (i.e. Samartzis classification scheme, level-specific and regional fusion patterns, sagittal and coronal alignment) were also assessed.

### Statistical analyses

SPSS (Chicago, IL) statistical software program was used to perform the statistical analysis. Descriptive and frequency statistics were performed on the data. Chi-square or Fisher’s exact tests were used for categorical variables where appropriate. Mann–Whitney-*U* test was used to assess the association between two groups. Univariate analysis was performed to assess the effect of the Samartzis et al. [7] KFS classification scheme upon the dependent variable of the presence of clinical triad findings, of which odds ratios (OR) and 95 % confidence intervals (CI) were obtained and assessed. If the data allowed, multivariate regression modeling was undertaken. The threshold for statistical significance was established at *p* < 0.05

## Results

**Fig. 1:**
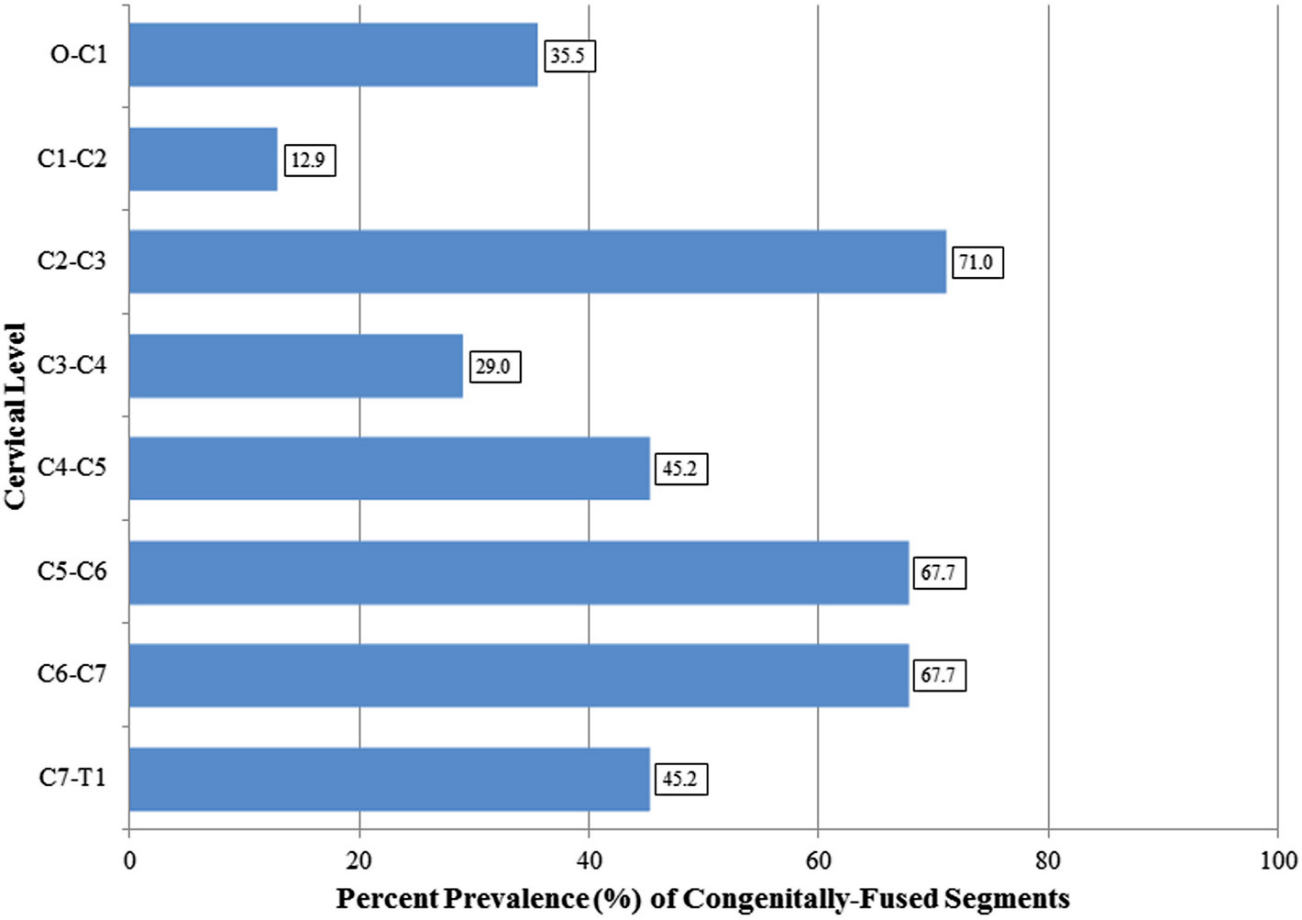
Bar graph illustrating the distribution of congenitally-fused cervical segments in KFS patients

There were 12 males (38.7 %) and 19 females (61.3 %) with a mean age at initial presentation of 9.7 years (range: 2 to 19, ±SD: 4.7 years). A mean of 3.4 congenitally-fused segments (range: 1 to 7, ±SD: 1.7 fused segments) was noted. The C2-C3 segment was the most commonly fused level, occurring in 22 patients (71 %) (Fig. 1). Occipitalization occurred in 11 patients (35.5 %). Based on the Samartzis et al. [7] KFS classification scheme, six (19.4 %) were Type I, 15 (48.4 %) were Type II, and 10 (32.3 %) were Type III. Out of 31 patients, congenital fusion consisted of various regions of the cervical spine (in isolation or in combination): the upper cervical region in 13 patients (41.9 %), the mid cervical region in 24 patients (77.4 %), and the lower cervical region in 25 patients (80.6 %). Eight (25.8 %), 15 (48.4 %), and 8 (25.8 %) patients demonstrated congenital fusion at only one, two, or three cervical regions, respectively. The mean coronal and sagittal cervical alignments were 19.1 degrees (range: 0 to 67, ±SD: 19.5 degrees) and 38.8 degrees (range: 14 to 64, ±SD: 15.7 degrees), respectively.

**Fig. 2:**
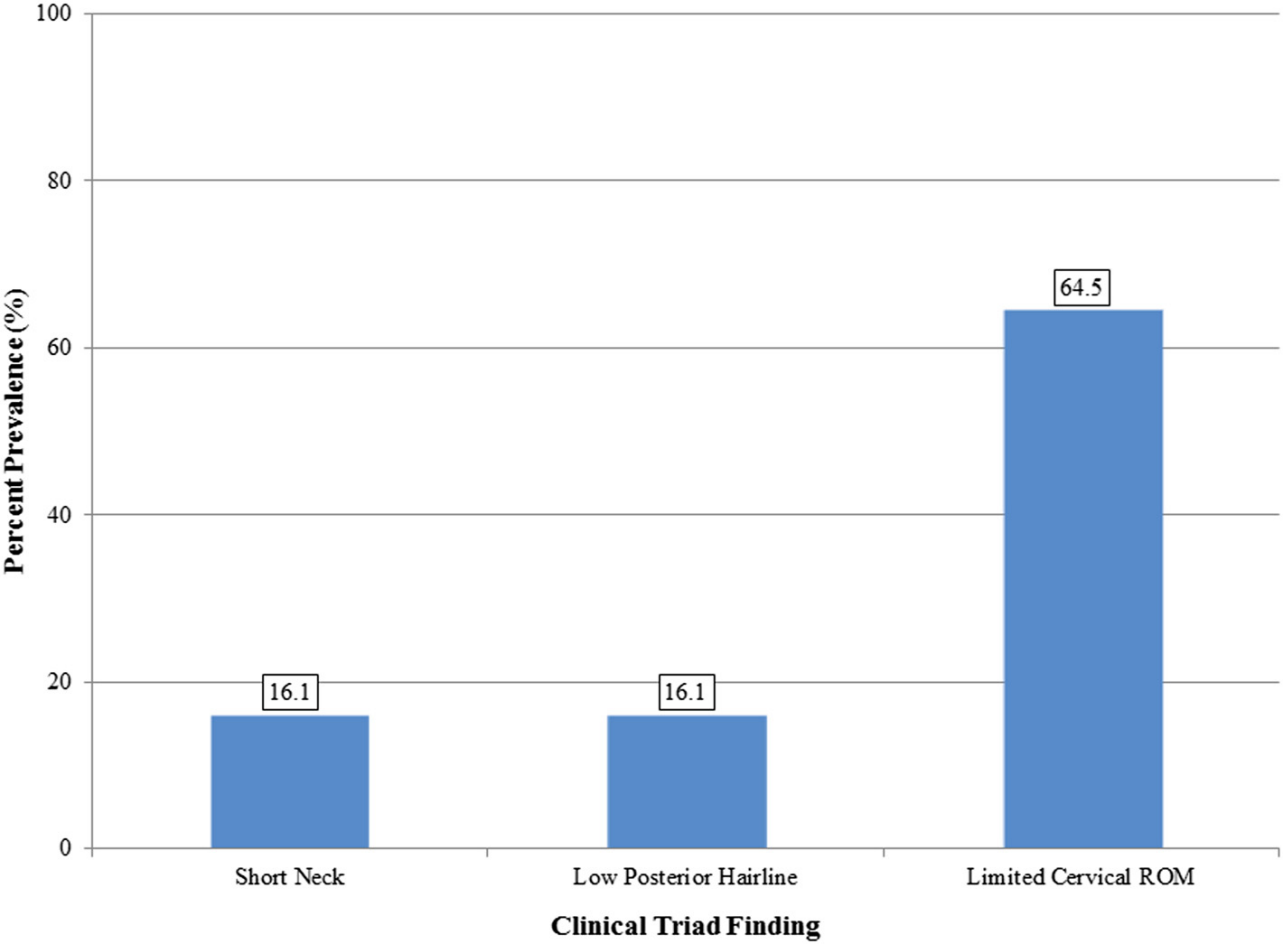
Bar graph showing the percent distribution of the clinical triad manifestation in KFS patients

**Fig. 3:**
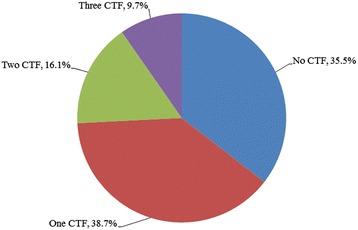
Pie chart illustrating the percent distribution of clinical triad manifestations in KFS patients. (CTF = clinical triad manifestation)

**Fig. 4:**
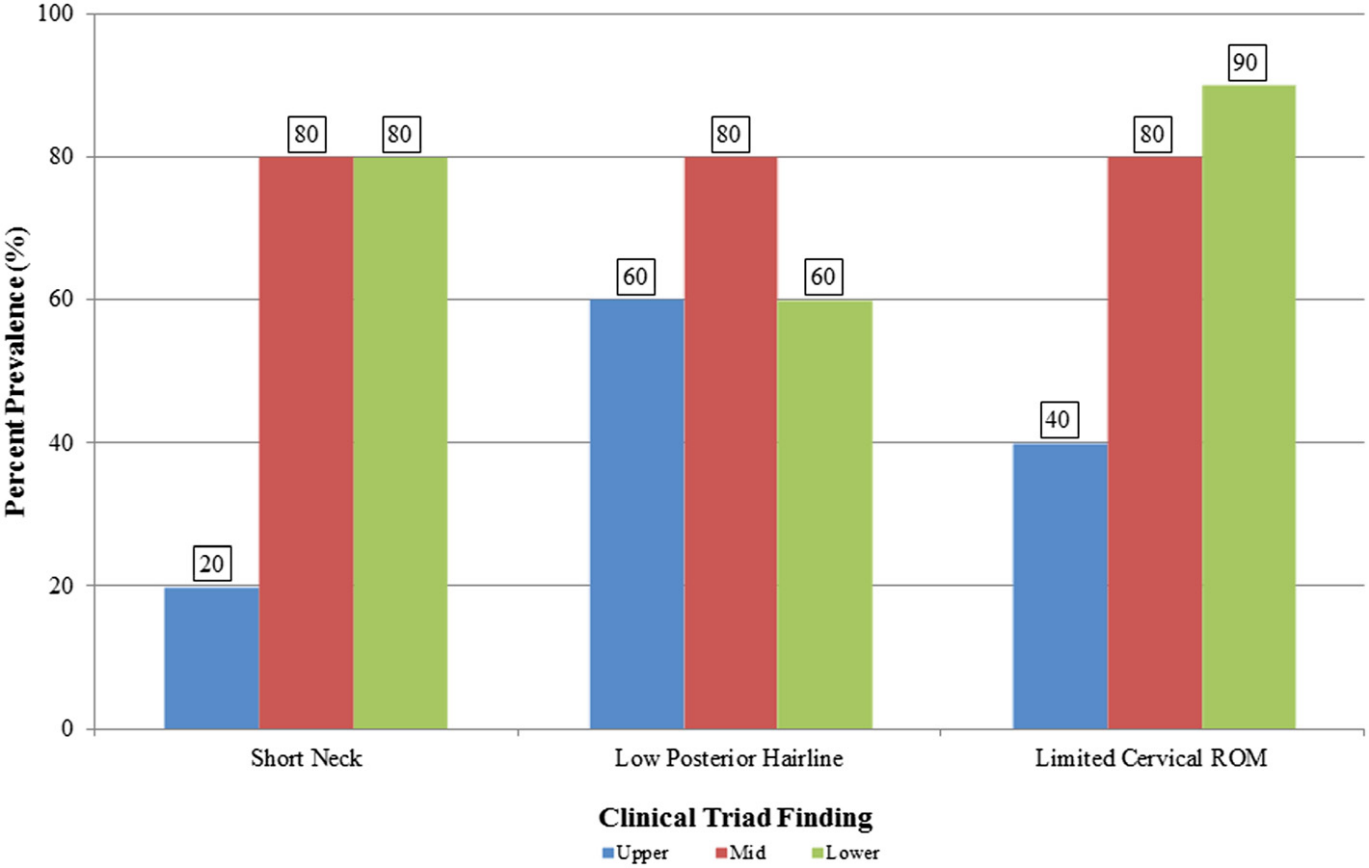
Bar graph demonstrating the percent prevalence of clinical triad findings in relation to the cervical spine region. (Upper = O-C2, Mid = C2-C4, Lower = C4-T1)

Of all subjects, five (16.1 %) exhibited short neck, five (16.1 %) presented with low posterior hairline, and 20 (64.5 %) had limited cervical ROM (Fig. 2). Eleven (35.5 %), 12 (38.7 %), five (16.1 %), and three (9.7 %) of the patients exhibited none or at least one, two, or all three clinical triad signs, respectively (Fig. 3). There was no statistically significant difference between sex-type, age, the presence of occipitalization, and coronal or sagittal alignments to any of the parameters regarding the clinical triad (*p* > 0.05). Table 1 illustrates the relation between the presence of various clinical triad findings and the number of congenitally-fused segments ranging from C1 to T1. Mann Whitney-U tests indicated that individuals who exhibited limited cervical ROM (*p* = 0.028) or at least one clinical triad finding (*p* = 0.028) were noted to have a significantly higher number of congenitally-fused segments than individuals without such findings. Exhibiting a short neck (*p* = 0.368), low posterior hairline (*p* = 0.891), two (0.056) or all three (*p* = 0.659) clinical triad findings did not demonstrate a statistically significant difference in relation to congenitally-fused segments. Although congenital fusion of the lower cervical spine presented with a high percent prevalence of the clinical triad signs, such observations in this sample size did not statistically differ between cervical regions (*p* > 0.05) (Fig. 4). In addition, there was no statistically significant difference with regards to any combination of congenitally-fused cervical regions to that of the clinical triad signs (*p* > 0.05).

**Table 1:** Patterns of clinical triad findings in relation to the number of fused cervical segments from C1 to T1 in KFS patients

	Number of Fused Cervical Segments, mean (range, ±SD)		
	No	Yes	*p*-value
Short Neck	n = 26, 3.3 (1–7, 1.7)	n = 5, 4.0 (1–6, 1.9)	*0.368*
Low Posterior Hairline	n = 26, 3.4 (1–7, 1.7)	n = 5, 3.4 (1–5, 1.8)	*0.891*
Limited Cervical ROM	n = 11, 2.5 (1–5, 1.6)	n = 20, 3.9 (1–7, 1.6)	*0.028* ^*a*^
One CTFs	n = 11, 2.5 (1–5, 1.6)	n = 20, 3.9 (1–7, 1.6)	*0.028* ^*a*^
Two CTFs	n = 26, 3.2 (1–7, 1.7)	n = 5, 4.6 (2–6, 1.5)	*0.056*
Complete CTFs	n = 28, 3.4 (1–7, 1.8)	n = 3, 3.0 (1–4, 1.7)	*0.659*

ROM: range of motion

CTFs: clinical triad findings

^a^ denotes statistical significance

**Fig. 5:**
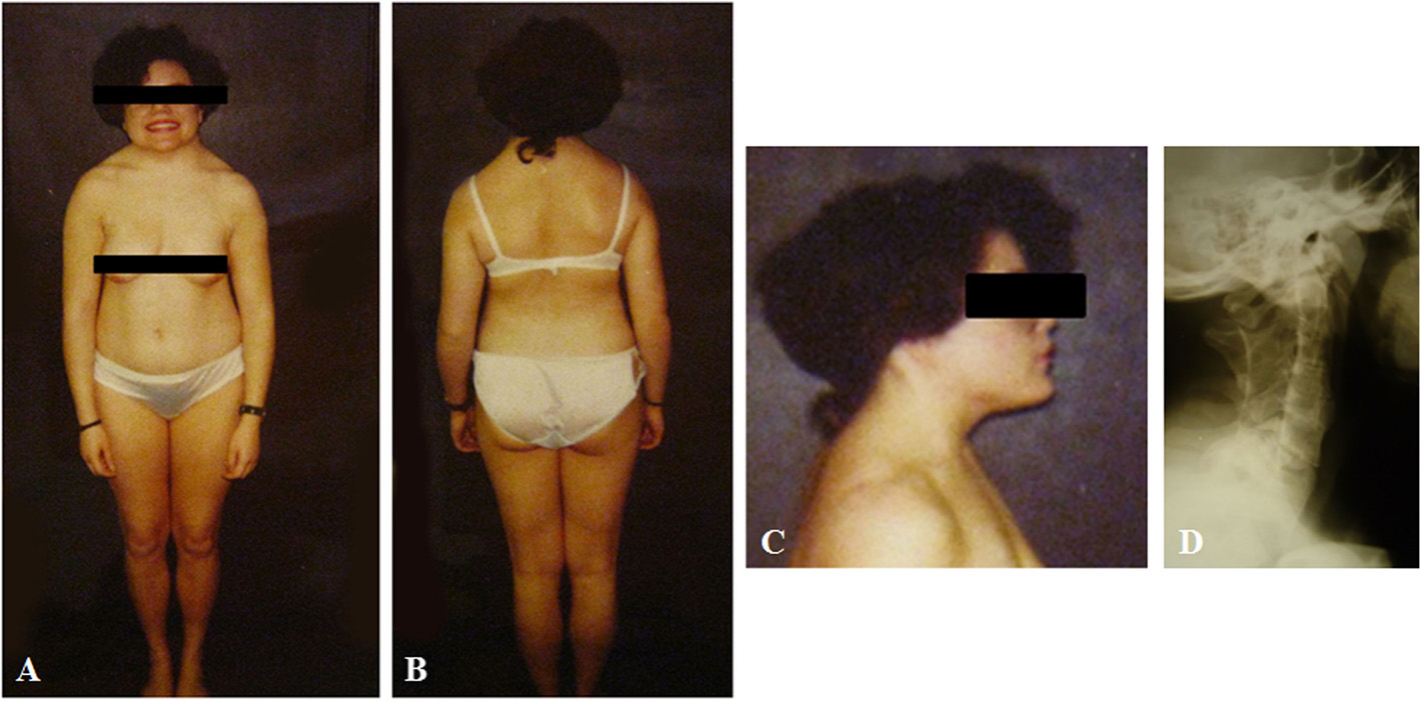
An 18-year-old KFS female with short neck, low posterior hairline, and limited cervical ROM. **a** Front, (**b**) back, and (**c**) lateral exterior views. **d** Lateral plain radiograph illustrating congenital fusion from C2-C6, exhibiting Samartzis et al. Type III classification

In our series, there were 20 patients (64.5 %) in Group 1 (with CFT signs) and 11 patients (35.5 %) in Group 2 (without CFT signs). Of the patients in Group 1, a short neck was noted in 25 %, a low posterior hairline was found in 30 %, and limited cervical ROM was found in all cases. Group 1 had a mean of 3.9 fused segments, whereas Group 2 had a mean of 2.5 fused segments (*p* = 0.028). There was no statistically significant difference between the age at presentation, sex-type, and occipitalization to Group-type (*p* > 0.05). In Samartzis et al. [7] KFS classification Types I, II, and III, there were 16.7, 73.3, and 80 %, respectively, in those who had at least one finding related to the clinical triad (i.e. Group 1). Univariate analysis noted that Samartzis et al. [7] KFS classification Type III presented with the highest likelihood of having any finding related to the clinical triad or limited cervical ROM (Type I reference: Type II OR: 13.8, 95 % CI: 1.20–156.5; Type III OR: 20.0, 95 % CI: 1.42–282.5) (Fig. 5). Due to the sample size and prevalence of clinical triad signs, multivariate modeling analysis was not practical.

## Discussion

Klippel-Feil syndrome was originally described in 1912 by Andre Klippel and Maurice Feil [2]. Their seminal report of this condition was based on a 46-year-old male French tailor who upon physical examination exhibited a short neck, low posterior hairline, and limited cervical ROM. Such findings have since become synonymous with the term “clinical triad” findings associated with KFS patients. Cadaveric assessment of their patient later on revealed complete improper segmentation or congenital fusion of the cervical spine.

Since the original description of congenital fusion of the cervical spine was reported by Klippel and Feil, segmentation errors involving any combination of fused cervical blocks have become tantamount with KFS. Such a condition can often alter the kinematics of the cervical spine that may accelerate degenerative changes throughout the region, cause hypermobility and instability leading to spinal cord injury, and may potentially lead to death [6, 7, 10, 13, 16, 18, 19, 27–35]. Patients with KFS may also have additional syndromes and anomalies [20]. Although various skeletal and extra-skeletal manifestations have been associated with KFS [1, 4, 20, 36], the most common associated manifestation is scoliosis, which may warrant surgical intervention [1, 9, 19, 20, 37]. As such, this has spawned interest among spine specialists throughout the years to better understand the heterogeneity of KFS and its clinical triad signs. For example, based on a retrospective study by Hensinger et al. [1] evaluating 50 KFS patients representing a mean age of 17 years at the time of assessment, the authors concluded that less than 50 % exhibited the clinical triad signs. In a retrospective study of 111 KFS patients with a mean age of 19 years, Pizzutillo and colleagues [19] noted a short neck, low posterior hairline, and limited cervical ROM in 41, 34, 76 % of the patients, respectively. According to the authors, the majority of KFS cases were normal in appearance and that detection of the congenitally-fused cervical segments was incidental upon plain radiographic assessment of the spine. In a retrospective study by Thomsen et al. [20] assessing 57 KFS patients with a mean age 27 years at follow-up, 74 % were noted to demonstrate a “short neck, often with low posterior hairline and a limitation of motion of the cervical spine.”

In our study examining 31 young KFS patients at a mean age of 9.7 years at initial assessment, 35.5 % did not exhibit any signs of the clinical triad; whereas, 38.7, 16.1, and 9.7 % exhibited one, two, or all three signs, respectively. The most predominant clinical triad finding in our study was limited cervical ROM, occurring in 64.5 % of the cases. As such, complete clinical triad findings were not commonly noted during the clinical assessment of young KFS patients. However, KFS patients with extensive, congenitally-fused segments were significantly more likely to exhibit one of the components of the clinical triad. We further investigated the effects of cervical fusion patterns by utilizing the KFS classification scheme as proposed by Samartzis et al. [7] that is specific to the cervical spine and that has shown to be clinically relevant for the development of cervical spine-related symptoms [7] and scoliosis [9]. Utilizing this scheme, our study noted that the clinical triad findings were more prevalent in KFS patients with a Samartzis et al. [7] Type III classification (multiple, contiguous fused segments). According to the literature, a Samartzis et al. [7] Type III classification represents the highest risk in developing radiculopathic or myelopathic symptoms.

The low prevalence of clinical triad findings in our study may be attributed to several reasons. Firstly, congenitally-fused cervical patterns in KFS are time dependent. Patients do not demonstrate their final fusion patterns from birth, but rather during adolescence and early adulthood. Therefore, the time of assessment may dictate the presence of any or all clinical triad signs because the underlying fusion process and pattern, coupled with growth and height development of an individual, may dictate the manifestation of clinical triad signs. If KFS patients are assessed later in life, as was the case for the studies by Hensinger et al. [1], Pizzutillo et al. [19], and Thomsen et al. [20], then the clinical triad signs may be more pronounced. Although in our current study of KFS patients we noted that age was not associated with any or all of the clinical triad signs, the majority of our sample population was relatively young with a mean age at initial presentation of 9.7 years. Secondly, clinical assessment pertaining to the clinical triad may be biased. Short neck may be a subtle finding and influenced by the height development of the patient. A low posterior hairline may not be visually striking and criteria to determine such a phenotype may vary between individuals. Nonetheless, KFS individuals with a short neck may concomitantly possess a short posterior hairline as was often noted in our study and in the reported literature. In a study by Samartzis et al. [12] assessing the vertebral dimensions of the cervical spine, the authors noted that congenital cervical fusion arrests normal appositional bone growth, but it remains unclear how the extent of fused segments may affect cervical height. Such findings may further explain variation upon the physical findings of short neck and low posterior hairline in KFS patients.

Numerous studies have noted that limited cervical ROM is the most predominant finding from the clinical triad signs [1, 19, 20]. Gray et al. [38] reported that patients with less than three fused cervical vertebrae or involvement of the lower cervical spine did not increase the risk of developing limited cervical ROM. In our current study, we noted a mean of four congenitally-fused cervical segments, which significantly increased the likelihood of noting such a sign. Furthermore, when assessing the effects of specific or combined congenitally-fused cervical regions and their association with any of the clinical triad signs, our study did not find any statistically significant effect. Considering that the sample size of this series is relatively low but rather large considering the infrequent nature of KFS in the general population, our study noted that congenital fusion of the lower cervical spine was more associated with certain clinical triad signs, particularly limited cervical ROM. Assessing this clinical phenotype based on the KFS classification scheme used, we noted that a Samartzis et al. [7] Type III patient presented with almost a 20-fold increased likelihood of having limited cervical ROM. It should be noted that since a Type III pattern entails multiple, contiguous congenitally-fused cervical segments, the likelihood of having three to four segments fused is very probable. In addition, it is also rather expected that as the number of congenitally-fused levels increases, this also alters the kinematics of the cervical spine and may potentially contribute to the phenotype of restricted motion.

The true incidence of KFS and its effects across populations remains unknown. It has been reported that KFS occurs in 1 out of every 42,000 births [21–24]. However, in a review of all the radiographic cervical spine films at a single hospital in Copenhagen, Gjorup et al. [39] noted that the incidence of KFS was 0.2 cases per 1000 individuals. According to Brown et al. [40] who assessed 1400 skeletons from the Terry collection in the United States, the incidence was 0.50 cases per 1000 skeletons. As such, the incidence of KFS may be much higher than what has been commonly purported. In general, KFS has typically been diagnosed incidentally upon radiographic examination and/or as part of a syndromic work-up, in particular for undergoing spine surgery for the operative management of congenital scoliosis. Until large population-based studies are undertaken to assess the incidence and etiology of KFS, an index of suspicion must be maintained, particularly in the setting of associated congenital spine conditions, wherein the cervical spine should be thoroughly assessed.

Klippel-Feil syndrome is a complex, congenital condition with substantial heterogeneity in phenotypic expression of spinal and extraspinal abnormalities. The etiology of KFS remains elusive and varied. Various studies have postulated that global fetal insult, vascular disruption, primary neural tube complications, or genetic factors may be responsible for the development of KFS [19, 22, 41–43]. The most common spinal finding in KFS patients is scoliosis [9, 18, 20, 34]. Advanced degenerative changes of the cervical spine causing stenosis [7, 18, 30, 34] or non-fused and unstable hypermobile segments [6, 13, 19, 44] present the greatest risk of neurologic compromise in KFS patients. Cervical spinal cord cross-sectional dimensions are also diminished in KFS individuals due to axonal loss or improper formation of the cord, which may increase the risk of neurologic compromise arising from a traumatic event or instability [27]. Thorough cervical spine evaluation is essential to avoid potential spinal cord injury stemming from laryngoscopy, intubation, intraoperative positioning and head manipulation that may increase the risk of craniovertebral dislocation and atlantoaxial subluxation [16, 35, 45]. Therefore, a missed diagnosis of KFS may potentially contribute to intraoperative complications and potential spinal cord injury.

Although our study broadens the understanding of KFS, there are several limitations. For one, the study is clinically retrospective in nature and, as such, the assessment of clinical triad signs may be biased, in particular since more than one spine specialist may have been involved in the original assessment of the patient. However, due to the infrequent nature of KFS cases presenting for consultation, it would be a challenge to perform a prospective study within a reasonable timeframe to address clinical triad signs at initial presentation because of the bias of expectation and recruitment issues. Such study design issues were also noted in all previous studies assessing clinical triad signs at initial presentation, which were all retrospective in nature. However, all patients in our study were consecutive, assessed at a single institution, and examined by the same spine team who underwent similar training. In order to minimize the effects of bias of interpretation of the clinical triad signs, the authors further analyzed the association of congenitally-fused cervical segments with at least one clinical triad sign. Secondly, the assessment of the presence of congenitally-fused cervical segments was performed based on plain radiographs, and advanced imaging (e.g. computed topography) was not utilized largely due to cost and to limit patient exposure to ionizing radiation. However, anteroposterior and dynamic views were utilized to assess the extent of fusion, and the prevalence of levels noted to exhibit congenital fusion were similar as other reported studies addressing KFS patients. Furthermore, although our study sample is relatively small to conduct meaningful multivariate modeling analysis on the main outcome, this unfortunately is a reflection of the uncommon incidence of this syndrome that hinders identification of relevant cases to facilitate further analysis. Also, no a priori size calculation was performed. However, in light of previous KFS studies, our series of 31 KFS cases is relatively substantial. Nonetheless, larger, prospective studies are needed to further validate our findings.

## Conclusions

In our series, the clinical triad findings of KFS in young patients are not common at initial clinical examination, which may result in under-reporting of the incidence of this condition. Limited cervical ROM was the most common finding of the clinical triad signs. The extent of congenitally-fused segments or Samartzis et al. Type III classification increases the association of exhibiting at least one of these clinical triad signs. The treating physician should maintain a high-index of suspicion for KFS and associated abnormalities, irrespective of clinical triad findings, especially in patients that exhibit associated spinal findings such as congenital scoliosis. Although this study is retrospective in nature and the sample size is relatively small but substantial given the infrequent nature of this condition, the purpose of this work is to raise awareness and to motivate additional discussion and research of the complex nature of KFS, especially regarding the clinical triad and physical manifestations that the literature may have adopted in haste, and propagated throughout the years without close scrutiny.
